# Protein biomarkers in cervicovaginal lavages for detection of endometrial cancer

**DOI:** 10.1186/s40364-022-00438-5

**Published:** 2022-12-02

**Authors:** Paweł Łaniewski, Haiyan Cui, Nichole D. Mahnert, Jamal Mourad, Matthew P. Borst, Lyndsay Willmott, Dana M. Chase, Denise J. Roe, Melissa M. Herbst-Kralovetz

**Affiliations:** 1 College of Medicine – Phoenix, University of Arizona, 425 N. 5th St, Phoenix, AZ 85004 USA; 2 UA Cancer Center, University of Arizona, 3838 N. Campbell Ave, Tucson, AZ 85719 USA; 3 Banner – University Medical Center, 1033 E. McDowell Rd, Phoenix, AZ 85006 USA; 4 Arizona Center for Cancer Care, 2222 E. Highland Ave, Phoenix, AZ 85016 USA

**Keywords:** Cervicovaginal microenvironment, Endometrial cancer, Minimally to non-invasive diagnostic, Protein biomarker, Uterine cancer, Women’s health

## Abstract

**Background:**

Rates of endometrial cancer (EC) are increasing. For a definitive diagnosis, women undergo various time-consuming and painful medical procedures, such as endometrial biopsy with or without hysteroscopy, and dilation and curettage, which may create a barrier to early detection and treatment, particularly for women with inadequate healthcare access. Thus, there is a need to develop robust EC diagnostics based on non- or minimally-invasive sampling. The objective of this study was to quantify a broad range of immuno-oncology proteins in cervicovaginal lavage (CVL) samples and investigate these proteins as predictive diagnostic biomarkers for EC.

**Methods:**

One hundred ninety-two women undergoing hysterectomy for benign or malignant indications were enrolled in this cross-sectional study. Classification of women to four disease groups: benign conditions (*n* = 108), endometrial hyperplasia (*n* = 18), low-grade endometrioid carcinoma (*n* = 53) and other EC subtypes (*n* = 13) was based on histopathology of biopsy samples collected after the surgery. CVL samples were collected in the operating room during the standard-of-care hysterectomy procedure. Concentrations of 72 proteins in CVL samples were evaluated using multiplex immunoassays. Global protein profiles were assessed using principal component and hierarchical clustering analyses. The relationships between protein levels and disease groups and disease severity were determined using Spearman correlation, univariate and multivariate receiver operating characteristics, and logistic regression analyses.

**Results:**

Women with EC and benign conditions exhibited distinctive cervicovaginal protein profiles. Several proteins in CVL samples (e.g., an immune checkpoint protein, TIM-3, growth factors, VEGF, TGF-α, and an anti-inflammatory cytokine, IL-10) discriminated EC from benign conditions, particularly, when tested in combinations with CA19–9, CA125, eotaxin, G-CSF, IL-6, MCP-1, MDC, MCP-3 and TRAIL (sensitivity of 86.1% and specificity of 87.9%). Furthermore, specific biomarkers (e.g., TIM-3, VEGF, TGF-α, TRAIL, MCP-3, IL-15, PD-L2, SCF) associated with histopathological tumor characteristics, including histological type and grade, tumor size, presence and depth of myometrial invasion or mismatch repair protein status, implying their potential utility for disease prognosis or monitoring therapies.

**Conclusions:**

This proof-of-principle study demonstrated that cervicovaginal sampling coupled with multiplex immunoassay technology can offer a minimally to non-invasive method for EC detection.

**Supplementary Information:**

The online version contains supplementary material available at 10.1186/s40364-022-00438-5.

## Background

Endometrial cancer (EC) is the most common gynecologic cancer and the fourth most common cancer affecting women in high-income countries [1]. In contrast to other malignancies, rates of EC continue to rise [2]. The International Agency for Research on Cancer estimates that EC rates will increase by more than 50% worldwide by 2040 [3]. Risk factors for EC include increased age, higher BMI, metabolic syndrome, estrogen exposure, tamoxifen use, early menarche, late menopause, lower parity, and genetic predisposition [4, 5]. There are also indications that social determinants of health and race/ethnicity contribute to risk and survival rates [6]. For example, in the USA, Black women with EC have an overall 55% higher 5-year mortality risk compared to White women, likely due to delayed diagnosis [7]. Hispanic and Native American women also have higher incidence and poorer survival rates of EC relative to non-Hispanic White women [8, 9]. In Europe, a Swedish study revealed that women with lower socioeconomic status are generally diagnosed at the late cancer stage and have reduced survival compared to women with higher socioeconomic status [10].

Historically, EC was grouped into two categories: type I (most common, estrogen-driven, composed of grade 1 or 2 endometrioid carcinomas with a favorable prognosis) and type II (less common, composed of high-grade endometrioid carcinomas or other non-endometrioid subtypes, more aggressive with a poor prognosis) [5]. Yet, EC are heterogenous at the molecular level. The new classification of EC into four molecular subgroups has been identified by The Cancer Genome Atlas Project [11]. These subgroups were defined by mutation burden and copy number alterations, including microsatellite instability with mismatch repair (MMR) defect, hypermutation of *POLE* gene, extensive genomic amplifications/deletions (copy number high) and low amount of genomic alterations (copy number low). Importantly, this and other molecular classifications allows subdividing EC into distinct prognostic groups, thus helping in determining treatment options and improving clinical outcomes [12].

EC is most often diagnosed in symptomatic women with abnormal uterine bleeding; however, this symptom is also common for other gynecologic conditions [5]. Currently, the gold standard for the diagnosis of EC is endometrial biopsy with or without hysteroscopy or dilation and curettage, which involves dilation of the cervix and scraping of the endometrial lining [13, 14]. Although these surgical procedures are considered to be minimally invasive and generally safe, they still carry risks of complications, including uterine perforation, uterine infection, and hemorrhage. In addition, current sampling methods for EC diagnosis can cause anxiety, physical discomfort and/or pain [15–18], which impact acceptability and accessibility. Thus, there is a need to develop a non-invasive and low-cost method for early EC detection.

Proteins are easily detectable and quantifiable in a variety of biological fluids, therefore commonly tested as potential biomarkers for cancer detection [19]. For EC, protein biomarkers have been mainly tested in blood or tissue samples [20, 21]. The two most studied proteins included human epididymis protein (HE4) and cancer antigen (CA) 125, both found elevated in endometrial tissues and serum of EC patients [22–26]. Although specific, these biomarkers (analyzed individually or in combination) failed to demonstrate high sensitivity [27–29]. Thus, additional research is needed to quantify protein biomarkers in the context of EC for sufficient diagnostic accuracy, preferably using samples collected by a non-invasive method.

Herein, we tested our hypothesis that the anatomical continuity of the female reproductive tract allows detection of EC-related protein biomarkers in the cervicovaginal microenvironment using minimally-invasive lavage sampling. To achieve this, we quantified a broad range of immuno-oncology biomarkers in cervicovaginal lavages (CVL), collected from women with EC and other gynecologic conditions, and investigated these proteins as predictive diagnostics for EC using biomarker discovery and machine learning algorithms. Overall, this proof-of-principle study shows that CVL sampling can offer a potential, minimally to non-invasive method for early detection of EC.

## Methods

### Study participants

Participants were recruited at three clinical sites located in Phoenix (AZ, USA) metropolitan area: Banner University Medical Center – Phoenix, Valleywise Health Medical Center, and Dignity Health Chandler Regional Medical Center between June 2018 and February 2020. One hundred ninety-two women undergoing hysterectomy for benign or malignant indications were enrolled and contributed to the study. Classification of women to four disease groups: benign conditions (*n* = 108), endometrial hyperplasia (*n* = 18), low-grade (grade 1 or 2) endometrioid carcinoma (EEC) (*n* = 53) and other EC (including grade 3 EEC or other histological subtypes) (*n* = 13) was based on histopathology of biopsy samples collected after the surgery. We included women of any race or ethnicity and aged 18 years or older. Exclusion criteria included: currently menstruating; currently lactating; currently on antibiotics, antifungals, antivirals or topical steroids; current vaginal infection (bacterial vaginosis, candidiasis), vulvar infection, urinary tract infection or sexually transmitted infection (chlamydia, gonorrhea, trichomoniasis, genital herpes) or within the previous 3 weeks; use of douching substances, vaginal medications, vaginal suppositories, feminine deodorant sprays, wipes, or lubricants within the previous 48 hours; use of depilatory treatments in the genital area within the previous 72 hours; any skin condition in the genital area interfering with the study; sexual intercourse within the previous 48 hours; bath or swimming within the previous 4 hours; smoking or consuming nicotine-contained products within the previous 2 hours; hepatitis; being HIV-positive. The exclusion criteria were verified by physician’s pelvic exam, medical record and/or self-reported. Demographic, socioeconomic, and medical history data were collected from surveys and/or medical records.

### Sample collection and processing

Clinical specimens were collected by a surgeon in the operating room during the standard-of-care hysterectomy procedure. All samples were obtained after anesthesia and prior to vaginal sterilization. Cervicovaginal lavage (CVL) samples were collected using a non-lubricated speculum and 10 ml of sterile 0.9% saline solution (Teknova, Hollister, CA). Following the collection, CVL samples were immediately placed on ice and frozen at − 80 °C within 1 hour. Prior to downstream analyses, CVL samples were thawed on ice, clarified by centrifugation (700×*g* for 10 min at 4 °C) and aliquoted to avoid multiple freeze-thaw cycles. All samples were stored at − 80 °C.

### Quantification of soluble proteins

Levels of 71 proteins (AFP, BTLA, CA15–3, CA19–9, CA125, CD27, CD28, CD40, CD80, CD86, CEA, CYFRA21-1, EGF, eotaxin/CCL11, Flt-3 L, FGF-2, fractalkine/CX3CL1, G-CSF, GITRL, GROα/CXCL1, GM-CSF, HE4, HGF, HVEM, ICOS, IFNα2, IFNγ, IL-1α, IL-1β, IL-2, IL-4, IL-5, IL-6, IL-7, IL-8/CXCL8, IL-9, IL-10, IL-12 (p40), IL-12 (p70), IL-13, IL-15, IL-17A, IP-10/CXCL10, LAG-3, leptin, MCP-1/CCL2, MCP-3/CCL7, MDC/CCL22, MIF, MIP-1α/CCL3, MIP-1β/CCL4, OPN, PD-1, PD-L1, PD-L2, PDGF-AA, PDGF-AB/BB, prolactin, PSA (total), RANTES/CCL5, SCF, sCD40L, sFas, sFasL, TGF-α, TIM-3, TLR2, TNFα, TNFβ, TRAIL, VEGF) were measured in CVL samples using the Milliplex MAP Magnetic Bead Immunoassays: Human Cytokine Chemokine Panel 1, Human Circulating Cancer Biomarker Panel 1 and Human Immuno-Oncology Checkpoint Protein Panel 1 (Millipore, Billerica, MA) in accordance with the manufacturer’s protocols. Data were collected with a Bio-Plex 200 instrument and analyzed using Manager 5.0 software (Bio-Rad, Hercules, CA). Levels of IL-36γ (IL-1F9) were measured in CVL samples by enzyme-linked immunosorbent assay using Human IL-36γ ELISA kit (RayBiotech, Norcross, GA) in accordance with the manufacturer’s instruction. A five-parameter logistic regression curve fit was used to determine the concentration. All samples were assayed in duplicate. The concentration values below the detection limit were substituted with 0.5 of the minimum detectable concentration provided in the manufacturer’s instructions. The logarithmic transformation was applied to normalize the data.

### Unsupervised data reduction analyses

The principal component analysis was performed to reduce the observed variables into a smaller number of principal components that account for most of the variance in the observed variables. For the first two principal components (PC1 and PC2), the difference among groups was assessed by the multivariate analysis of variance model. The statistical differences for individual components were assessed using an analysis of variance. If the overall difference was significant (*P* < 0.05), pairwise comparisons with Bonferroni adjustment were performed. The hierarchical clustering analysis was performed to show relationships of protein biomarker levels to metadata available for each patient, i.e., disease group, menopausal status, and BMI. Prior to clustering, levels of each protein biomarker were mean centered and then variance was scaled. Hierarchical clustering was performed using ClustVis server [30] and based on Euclidean distance and Ward linkage. The statistical differences in distribution of patient-related factors between clusters were assessed using Fisher’s exact test or chi square test.

### Receiver operating characteristics (ROC) analysis

The univariate ROC analysis was performed to identify protein biomarkers that discriminate specific disease groups with high sensitivity and specificity. The mean levels of proteins for each patient were used in the analyses. The strength of the discriminators was measured with area under the curve (AUC) values. Proteins with AUC greater than or equal to 0.8, or 0.9 were considered as good, or excellent discriminators, respectively.

### Supervised machine-learning analyses

Supervised learning was performed using the logistic regression algorithm. The features were selected based on the least absolute shrinkage and selection operator (LASSO) modelling. The performance of the predictive model was evaluated using the Monte Carlo cross-validation, which uses 2/3 of samples for model training and the remaining 1/3 of samples for testing. One hundred cross validations were performed, and the results were averaged to generate plots. Evaluation metrics included the AUC of multivariate ROC analysis and the confusion matrix calculated at a probability threshold of 0.5. The analysis was performed using MetaboAnalyst 5.0 [31].

### Volcano plot and correlation analyses

Differences in the protein biomarker levels among patients diagnosed with EC stratified based on histological type and grade, tumor size, presence of myometrial invasion, and MMR status were tested using multiple *t* tests and corrected using false discovery rate (FDR) method. Differences in mean protein levels and *q* values were graphically presented as volcano plots. Protein biomarkers with *q* < 0.1 were considered significant. The Spearman’s rank correlation analysis was also performed to investigate the association of protein biomarker levels with the tumor size (measured in cm) and the depth of myometrial invasion (measured in mm). A correlation matrix was computed using correlation coefficients (r) with *P* values, and graphically presented as a heat map. *P* < 0.05 was considered significant.

### Other statistical analyses

Differences in the demographic, socioeconomic and other patient-related variables between disease groups were tested using the Kruskal-Wallis test for continuous variables and Fisher’s exact test for categorical variables. The statistical differences in the concentrations of protein biomarkers among the patient groups were tested using a linear mixed effects model where the group was a fixed effect and the replicate was the random effect. If the overall difference was significant (*P* < 0.05), paired tests were performed with Bonferroni adjustment. Comparisons were adjusted for age and BMI in the linear mixed effects models by including these variables as predictors in the models, in addition to indicators for the patient groups (with benign as the reference group). Statistical analyses were performed using SAS 9.4 (SAS Institute, Cary, NC) unless otherwise indicated.

## Results

### Study population

A total of 192 women undergoing hysterectomy were recruited and enrolled in this cross-sectional study (Table 1). Women were classified into four disease groups: benign conditions (*n* = 108), endometrial hyperplasia (*n* = 18), low-grade endometrioid carcinoma (EEC) (*n* = 53), and other EC subtypes (*n* = 13). The classification into groups was based on histology of biopsy samples. The detailed patient demographics and characteristics are included in Supplementary Table S1. The average age and body mass index (BMI) were 51 years and 34.8 kg/m^2^, respectively. Regarding race and ethnicity, participants were predominantly Caucasian (74.7%) with a relatively high proportion of women identifying as Hispanic (26.2%). Women diagnosed with low-grade EEC and other EC subtypes were older (mean 58.7 and 60.8 years, respectively) compared to women diagnosed with benign conditions 45.6 years; *P* < 0.0001) and mostly postmenopausal (76.5 and 92.3% vs. 17.6%; *P* < 0.0001). Women with low-grade EEC had also higher body mass index (BMI; mean 40.3 kg/m^2^) than women with benign conditions (mean 30.6 kg/m^2^; *P* < 0.0001). In addition, there were significant differences in other comorbidities, such as diabetes (*P* = 0.006) and hypertension (*P* = 0.002) among the groups; however, these differences were attenuated after controlling for BMI or age (Supplementary Table S2).

**Table 1:** Patient demographics. Race and menopause status data were available for 190 women; ethnicity data were available for 191 women. *P* values were calculated using Kruskal-Wallis test for continuous variables and Fisher’s exact test for categorical variables

	All (*n* = 192)	Benign conditions (*n* = 108)	Endometrial hyperplasia (*n* = 18)	Low-grade endometrioid carcinoma (*n* = 53)	Other endometrial cancer subtypes (*n* = 13)	***P*** value
**Age** [mean (SD)]	51.02 (12.45)	45.55 (10.01)	54.11 (13.35)	58.73 (11.82)	60.77 (8.06)	< 0.0001
**Race** [*n* (%)]						
White/Caucasian	142 (74.74)	78 (72.90)	16 (88.89)	37 (71.15)	11 (84.62)	0.16
American Indian/Alaska Native	15 (7.89)	5 (4.67)	1 (5.56)	8 (15.38)	1 (7.69)	
Black/African American	12 (6.32)	11 (10.28)	0 (0.00)	1 (1.92)	0 (0.00)	
Other	21 (11.05)	13 (12.15)	1 (5.56)	6 (11.54)	1 (7.69)	
**Ethnicity** [*n* (%)]						
Hispanic	50 (26.18)	32 (29.63)	4 (22.22)	11 (21.15)	3 (23.08)	0.67
Non-Hispanic	141 (73.82)	76 (70.37)	14 (77.78)	41 (78.85)	10 (76.92)	
**Body mass index** [mean (SD)]	34.76 (10.16)	30.63 (7.54)	41.49 (7.45)	40.29 (11.07)	37.22 (12.76)	< 0.0001
**Menopause status** [*n* (%)]						
Premenopausal	108 (56.84)	89 (82.41)	6 (33.33)	12 (23.53)	1 (7.69)	< 0.0001
Postmenopausal	82 (43.16)	19 (17.59)	12 (66.67)	39 (76.47)	12 (92.31)	

### Cervicovaginal protein profiles

**Fig. 1:**
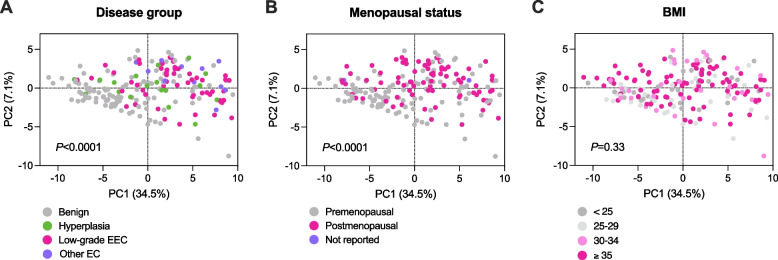
Women diagnosed with EC exhibit distinct cervicovaginal protein profiles compared to women with benign conditions. A principal component analysis (PCA) of 72 proteins in cervicovaginal lavages (*n* = 192) displayed along the first two principal components (PC). Each point represents a single sample colored based on disease group (**A**), menopausal status (**B**), or body mass index (BMI) (**C**). Significant differences among the disease groups and between pre- and postmenopausal women were assessed using a multivariate analysis of variance (MANOVA) model

CVL samples were collected from all participants (*n* = 192) and used to quantify 72 soluble proteins, including cytokines, chemokines, growth factors, apoptosis-related proteins, hormones, circulating tumor markers, and immune checkpoint proteins (see Materials and Methods). All tested proteins were measurable in CVL. The principal component analysis (PCA) was used to illustrate global protein profiles of individual samples (Fig. 1). PCA reduces the dimensionality of large datasets while preserving the maximum information amount. A set of variables (i.e., cervicovaginal levels of 72 protein) were transformed to a smaller number of principal components that account for most of the variance. We utilized the first two principal components (PC1 and PC2), which explained 41.6% of the variance in the data. A multivariate analysis of variance revealed significant differences among the disease groups (*P* < 0.0001) (Fig. 1A). The analysis also demonstrated that global protein profiles significantly differ between premenopausal and postmenopausal women (*P* < 0.0001) (Fig. 1B), but not between women varying by BMI (*P* = 0.33) (Fig. 1C). Subsequent pairwise comparisons showed that PC1 significantly varied between the disease groups (low-grade EEC vs. benign, *P* < 0.0001; other EC vs. benign, *P* < 0.0001) and menopausal status (*P* = 0.001) but did not vary among the BMI categories (Supplementary Fig. S1). PC2 was also significantly different between the disease groups (other EC vs. benign, *P* = 0.02) and the menopausal status categories (*P* < 0.0001).

**Fig. 2:**
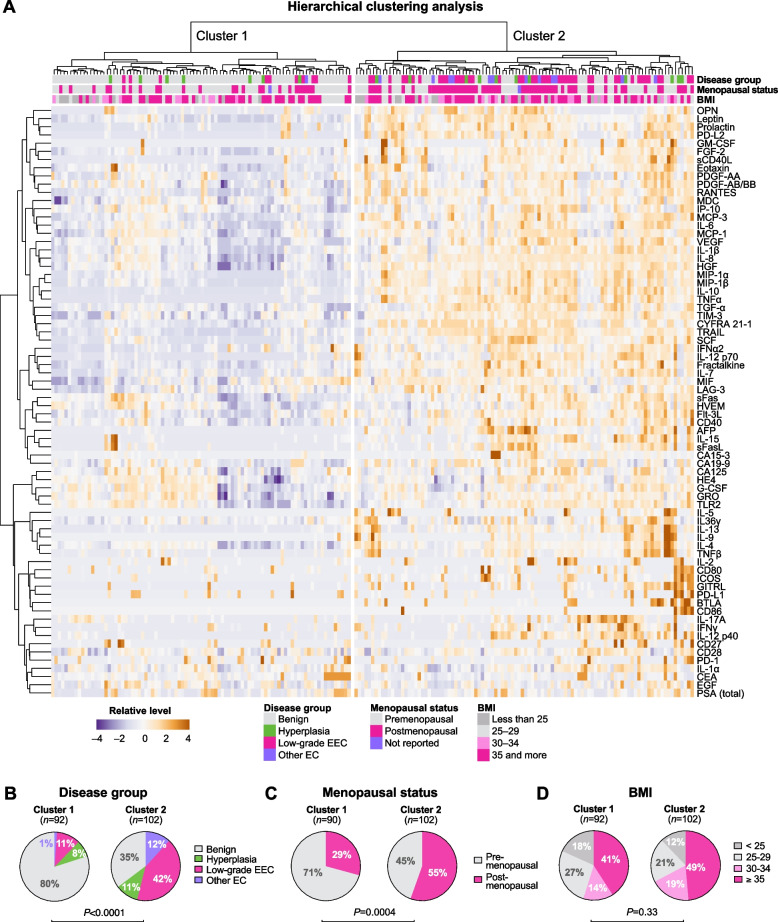
Cervicovaginal protein levels associates with the disease groups and menopausal status. A heatmap reflects relative levels of proteins in cervicovaginal lavages (CVL) across all the samples (*n* = 192) (**A**). Data were mean-centered and variance scaled along each row before clustering. A hierarchical clustering was based on Euclidean distance and Ward linkage. The analysis revealed two distinct clusters. Pie charts show distribution of the disease groups (**B)**, menopausal status (**C**) and BMI categories (**D**) were significantly different between the clusters. BMI categories did not vary between the clusters. *P* values were calculated using Fisher’s exact test or chi square test

To further analyze global protein profiles, we performed an unsupervised hierarchical clustering analysis (Fig. 2). A heatmap with a dendrogram revealed two distinct clusters. To characterize these clusters, we plotted the metadata (such as disease group, menopausal status and BMI) related to individual samples above the heatmap (Fig. 2A) and analyzed statistical differences among these patient-related factors between the clusters. The distribution of disease groups significantly varied (*P* < 0.0001) between the clusters. Cluster 1 was predominated by samples from the benign group (80%), whereas the cluster 2 had the highest proportion of samples from women diagnosed with EC (64%) (Fig. 2B). The menopausal status also significantly varied between the clusters (*P* = 0.0004) (Fig. 2C); however, there were no differences in distribution of BMI categories (*P* = 0.33) (Fig. 2D). Overall, the data reduction analyses revealed that women with benign conditions and women with EC exhibit distinctive cervicovaginal protein profiles.

### Cervicovaginal biomarkers for detection of EC

**Fig. 3:**
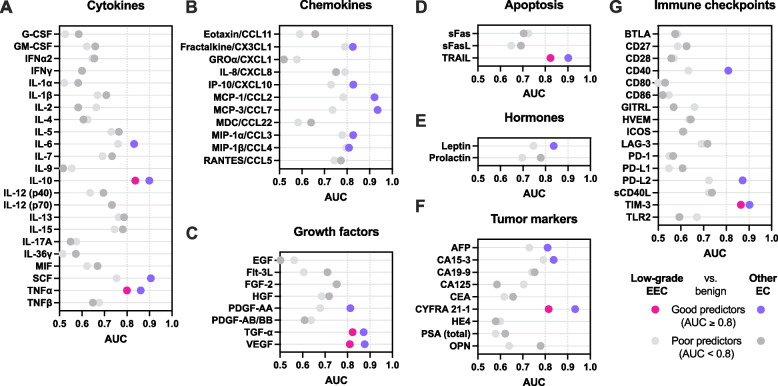
Protein biomarkers in cervicovaginal lavages discriminate patients diagnosed with EC from patients with benign conditions. Cervicovaginal biomarkers discriminating low-grade endometrial endometrioid carcinoma (EEC) or other endometrial cancer (EC) subtypes from benign conditions were identified using the receiver operating characteristics (ROC) analysis. The area under the curve (AUC) was reported for each tested protein, including cytokines (**A**), chemokines (**B**), growth factors (**C**), apoptosis-related proteins (**D**), hormones (**E**), tumor markers (**F**), and immune checkpoint proteins (**G**). The strength of the discriminators was measured with AUC values. Proteins with AUC greater than or equal to 0.8 or 0.9 (indicated in pink or purple) were considered as good or excellent discriminators, respectively

**Fig. 4:**
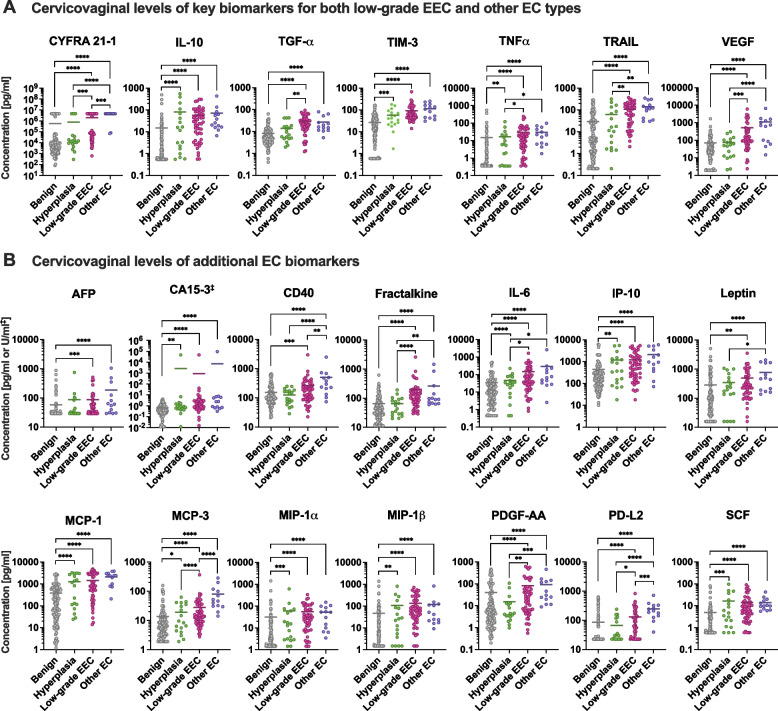
Key cervicovaginal biomarkers are elevated in both low-grade endometrioid carcinoma and other EC subtypes. Cervicovaginal levels of proteins, identified as good biomarkers for both low-grade EEC and other EC subtypes (**A**) or just for other EC subtypes (**B**) in the ROC analysis. Scatter dot plots show concentrations of these proteins in individual samples among the disease groups. A horizontal line indicates the mean. The significant differences were assessed using linear mixed effects models with Bonferroni adjustment. Asterisks indicate *P* values adjusted (* *P* < 0.05; ** *P* < 0.01; *** *P* < 0.001; **** *P* < 0.0001)

Next, we compared the levels of proteins measured in CVL samples among the disease groups. Since age and BMI were significantly different among the disease groups (Table 1), we adjusted *P* values for these factors. Fifty-four out of 72 protein targets were significantly elevated in women with low-grade EEC compared to benign (*P* ranging from 0.05 to < 0.0001) (Supplementary Table S3). We also found 20 targets significantly elevated in endometrial hyperplasia (*P* ranging from 0.02 to < 0.0001) and 40 targets elevated in other EC subtypes (*P* ranging from 0.05 to < 0.0001) (Supplementary Table S3). To identify biomarkers with high sensitivity and specificity, we performed a receiver operating characteristics (ROC) analysis. Proteins with the area under the curve (AUC), which shows the relationship between sensitivity and specificity, greater than or equal to 0.8 were considered as good discriminators. The analysis comparing low-grade EEC or other EC to benign conditions revealed seven proteins with good discriminatory properties for both EC subtypes: TIM-3 (AUC 0.86 and 0.90), IL-10 (AUC 0.84 and 0.90), TRAIL (AUC 0.82 and 0.90), TGF-α (AUC 0.82 and 0.87), CYFRA 21-1 (AUC 0.82 and 0.93), VEGF (AUC 0.81 and 0.88), and TNFα (AUC 0.80 and 0.86) (Fig. 3A-G and Supplementary Fig. S2). Notably, all seven proteins reached higher AUC values for the other EC group than for the low-grade EEC group. In addition, the analysis revealed 14 additional proteins (including cytokines: IL-6 and SCF, chemokines: fractalkine, IP-10, MCP-1, MCP-3, MIP-1α, and MIP-1β, a growth factor PDGF-AA, a hormone leptin, tumor markers: AFP, CA15–3, and immune checkpoint proteins: CD40 and PD-L2) with good discriminatory properties for other EC subtype group, but not low-grade EEC when compared to benign conditions (Fig. 3). When the cervicovaginal levels of key biomarkers for both EC subtypes, identified in the ROC analysis, were compared among the disease groups, all seven proteins (CYFRA 21-1, IL-10, TGF-α, TIM-3, TNFα, TRAIL and VEGF were significantly (*P* < 0.0001) elevated in both low-grade EEC and other EC groups when compared to benign conditions (Fig. 4A). Of those, only CYFRA-21 significantly (*P* = 0.001) differed in mean levels between low-grade EEC and other EC subtypes. In addition, IL-10 and TIM-3 levels were also elevated in endometrial hyperplasia patients when compared to the benign group (*P* < 0.0001 and *P* = 0.006, respectively). For additional 14 biomarkers for other EC, identified in ROC analysis, cervicovaginal levels were significantly elevated also in both EC groups (low-grade EEC and other EC subtypes) when compared to benign conditions (*P* ranging from 0.001 to < 0.0001). Three out of 14 biomarkers: CD40, MCP-3, and PD-L2 had higher cervicovaginal levels in other EC than low-grade EEC group (*P* ranging from 0.008 to < 0.0001). Notably, MCP-3 was also identified as a good discriminator (AUC = 0.832) between other EC subtypes and low-grade EEC in the subsequent ROC analysis (Supplementary Figs. S2 and S3). In addition, levels of eight proteins, mostly chemokines, i.e. CA15–3, IL-6, IP-10, MCP-1, MCP-3, MIP-1α, and MIP-1β were significantly (*P* ranging from 0.01 to < 0.0001) elevated in the endometrial hyperplasia group when compared to patients with benign conditions. Overall, these analyses identified potential biomarker candidates for the detection of EC using CVL sampling.

### Machine-learning modeling to predict EC

**Fig. 5:**
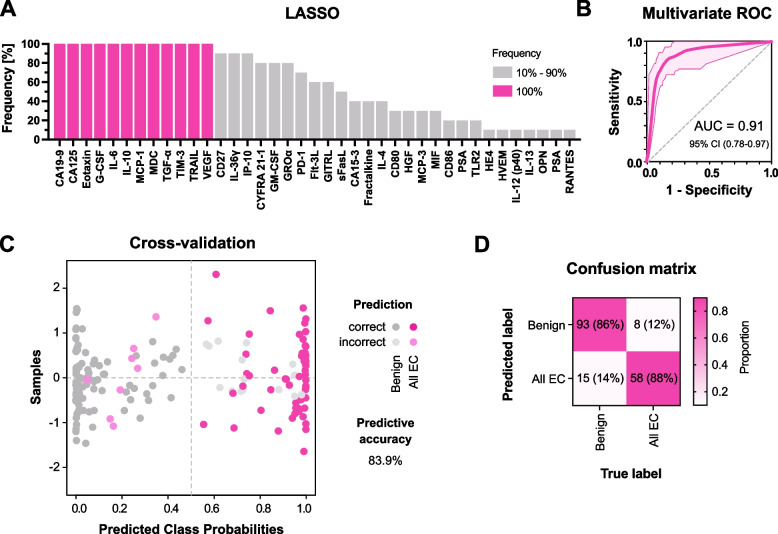
The logistic regression model accurately predicts EC and benign conditions using protein biomarkers in CVL samples. The least absolute shrinkage and selection operator (LASSO) was performed to select features to build the logistic regression model (**A**). Twelve proteins with 100% frequency of LASSO selection were used to build the model. The performance of the model was evaluated using the Monte Carlo cross-validation. A multivariate ROC analysis, showing true and false positive rates, indicates excellent prediction of EC when compared to benign conditions (AUC 0.91) (**B**). A scatterplot depicts the predicted class probabilities of all samples using the classifier at a threshold of 0.5 (**C**). The confusion matrix illustrates the proportion of times each sample receives the correct classification (**D**). The logistic regression model correctly classified 151 out of 174 tested samples (86.8%)

To evaluate the ability of multiple cervicovaginal protein biomarkers to predict the disease group (all EC subtypes vs. benign conditions), we used the logistic regression classification with the Monte Carlo cross-validation (Fig. 5). The predictive model was built using 12 protein biomarkers with 100% frequency in the least absolute shrinkage and selection operator method (i.e., CA19–9, CA125, eotaxin, G-CSF, IL-6, IL-10, MCP-1, MDC, TGF-α, TIM-3, TRAIL, and VEGF) (Fig. 5A). Five out of 12 biomarkers (IL-10, TGF-α, TIM-3, TRAIL, and VEGF) exhibited good discriminatory properties (AUC > 0.8) for both EC subtype groups when compared to benign conditions, and three biomarkers (IL-6, MCP-1, and MDC) exhibited good discriminatory properties for other EC subtype, but not for the low-grade EEC group, in the previous univariate ROC analysis (Fig. 3). In a subsequent multivariate ROC analysis, the model based on the selected 12 biomarkers demonstrated an excellent ability to discriminate patients with EC and benign conditions (average AUC 0.91) (Fig. 5B). Overall, the average predictive accuracy of the model based on 100 cross-validations was 83.9% (Fig. 5C). We also created a confusion matrix to show the proportion of time each sample obtained correct classification (Fig. 5D). Ninety-three out of 108 benign samples were correctly classified (sensitivity of 86.1%) and 58 out of 66 EC samples were correctly classified using the model (specificity of 87.9%). Overall, this analysis showed that coupling multiple cervicovaginal biomarkers with machine learning algorithms can increase the ability of created models to accurately predict the disease group.

### Cervicovaginal proteins and the severity of EC

**Fig. 6:**
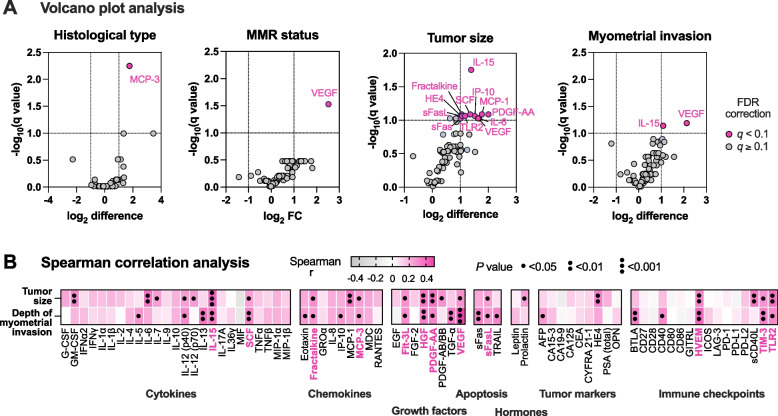
Cervicovaginal levels of protein biomarkers in patients with EC vary based on histological type, MMR status, tumor size, and myometrial invasion. A volcano plot analysis was used to assess differences in the protein levels among patient with EC stratified based on tumor characteristics, including histological type and grade, mismatch repair (MMR) protein expression, tumor size, and presence of myometrial invasion (**A**). Statistical significance was determined using multiple *t*-test with the false discovery rate (FDR) correction. A volcano plots indicate log_2_ differences (*x* axis) and -log_10_(*q* value) (*y* axis). Proteins with *q* < 0.01 were considered significant. A correlation analysis between cervicovaginal levels of 72 proteins with the tumor size (measured in cm) and the depth of myometrial invasion (measured in mm) (**B**). Correlation coefficients (r) were calculated using the Spearman’s rank correlation and depicted as a heatmap. *P* values are indicated with black circles. The biomarkers identified to be significant in both volcano and correlation analyses are marked with an asterisk (*)

To identify relationships between the cervicovaginal biomarker levels and the severity of EC, we extracted data from pathology reports on FIGO stage, histological type and grade, tumor size, presence and depth of myometrial invasion, presence of lymphovascular invasion, and mismatch repair (MMR) protein expression (Table 2 and Supplementary Fig. S5). Out of 66 women diagnosed with EC, 59 (89.4%) had endometrioid carcinomas or adenocarcinomas. The majority of EC (87.1%) were stage I tumors, were of low grade (i.e., grade 1 or 2; 86.4%) and had size greater than 2 cm (70%) (Supplementary Fig. S4). Myometrial and lymphovascular invasion were present in 69.7 and 6.2% of EC tumors, respectively. The MMR deficiency (i.e., loss of MLH1, PMS2, MSH2 or MSH6 expression) was observed in 23.3% of EC tumors. We categorized EC tumors based on histological type (low-grade EEC vs. other EC subtypes), MMR status (MMR-deficient vs. MMR-proficient), size (≤2 cm vs. > 2 cm), and presence of myometrial invasion and compared the cervicovaginal levels of protein biomarkers between these subgroups (Fig. 6A and Supplementary Fig. S5). We did not analyze FIGO stage or lymphovascular invasion due to unbalanced distribution of these characteristics among our cohort (Table 2). Following the false discovery rate correction for multiple comparisons (*q* < 0.1), the analysis revealed that only one protein, MCP-3, was significantly elevated in other EC subtypes when compared to low-grade EEC. When tumors were stratified based on MMR status, VEGF was significantly elevated in CVL samples from patients with MMR-deficient EC. Furthermore, cervicovaginal levels of 12 proteins (fractalkine, HE4, IL-6, IL-15, IP-10, MCP-1, PDGF-AA, sFas, sFasL, SCF, TLR2, VEGF) were significantly elevated in patients with larger tumors (> 2 cm) compared to patients with smaller tumors (≤2 cm). IL-15 and VEGF also levels varied between groups stratified based on the presence of myometrial invasion. In addition, we perform a correlation analysis between levels of proteins in CVL and size of tumors (measured in cm) and depth of myometrial invasion (measured in mm) (Fig. 6B, Supplementary Figs. S6 and S7). Twelve protein markers (cytokines: IL-15 and SCF; chemokines: fractalkine and MCP-3; growth factors: Flt-3 L, HGF, PDGF-AA, and VEGF; an apoptosis-related protein, sFasL; and immune checkpoint proteins: HVEM, TIM-3, and TLR2) significantly correlated with both tumor size and depth of myometrial invasion. Notably, four biomarkers identified in the correlation analysis (TIM-3, VEGF, TGF-α, and TRAIL) were highly discriminatory for both low-grade EEC and other EC subtypes (Fig. 3). Among them, TIM-3 and VEGF associated with tumor size, myometrial invasion, and MMR status, whereas TGF-α and TRAIL levels associated with myometrial invasion, but not other tumor characteristics. Overall, this analysis revealed that cervicovaginal sampling of protein biomarkers can allow for detection of EC detection and stratification of patients based on tumor characteristics.

**Table 2:** Characteristics of EC tumors in our cohort. Data on histological type, FIGO stage, tumor grade, tumor size, presence and depth of myometrial invasion, presence of lymphovascular invasion and MMR protein status were extracted from pathology reports. *n* indicates data availability

Characteristics		***n*** (%)
**Histological type** (*n* = 66)	Endometrioid adenocarcinoma	33 (50.0)
	Endometrioid carcinoma	26 (39.4)
	Serous carcinoma	4 (6.1)
	Other	3 (4.5)
**FIGO stage** (*n* = 62)	I	1 (1.6)
	IA	43 (69.4)
	IB	10 (16.1)
	II	2 (3.2)
	IIIC	4 (6.5)
	IV	2 (3.2)
**Tumor grade** (*n* = 66)	Grade 1	42 (63.6)
	Grade 2	15 (22.7)
	Grade 3	9 (13.6)
**Size** (*n* = 62)	≤ 2 cm	17 (27.4)
	> 2 cm	45 (72.6)
**Myometrial invasion** (*n* = 66)	no	20 (30.3)
	yes	46 (69.7)
	depth reported	40 (60.6)
	depth not reported	6 (9.1)
**Lymphovascular invasion** (*n* = 65)	no	61 (93.8)
	yes	4 (6.2)
**MMR protein status** (*n* = 60)	MMR-proficient	46 (76.7)
	MMR-deficient	14 (23.3)
Loss of nuclear expression	MLH1	9 (15.0)
	PMS2	11 (18.3)
	MSH2	1 (1.7)
	MSH6	4 (6.7)

## Discussion

Typically, EC is diagnosed in peri- and postmenopausal women with abnormal uterine bleeding [5]. Although this symptom is incredibly common in EC patients (occurs in approximately 90% cases) [32], only 9% of women with abnormal uterine bleeding are actually diagnosed with EC [32]. For a definitive diagnosis of EC, symptomatic women undergo various painful, anxiety-provoking and time-consuming medical procedures, such as hysteroscopy, endometrial biopsy, and dilation and curettage [13, 14]. This diagnostic approach forms a barrier to early detection and treatment, particularly in populations with limited or inadequate access to healthcare [6]. Thus, novel diagnostic methods, ideally based on non- to minimally invasive sampling, are needed to improve detection, increase acceptability, and ultimately reduce morbidity and mortality related to this common gynecological cancer.

Herein, we exploited lavage sampling of the cervicovaginal microenvironment coupled with multiplex immunoassay technology and demonstrated the utility of this novel approach for detection of EC. To our knowledge, this is the first study investigating protein biomarkers in CVL specimens for EC diagnosis. Other biological specimens that have been previously evaluated for detection of EC include endometrial tissues, uterine lavages/aspirates, blood, and urine [20]. Notably, of those specimens, only blood and urine samples, similarly to CVL, are collected by minimally or non-invasive means. While protein biomarkers in serum have been investigated for EC diagnosis since the 1980s [33], there are still no current serologic markers for clinical use. It is likely that quantities of EC-related proteins in circulation are too low, especially at the early phase of disease, limiting their diagnostic potential. To date, only a few studies investigated protein biomarkers in urine samples [34–36], but similarly to blood this approach has limitations as it relies on detection of circulating biomarkers. In contrast, due to the anatomical continuity of the female reproductive tract, cervicovaginal fluids might contain high concentrations of EC-related proteins. Thus, CVL sampling, as demonstrated in this study, can offer an attractive, non- to minimally invasive method of EC diagnosis. Previously, liquid from the Papanicolaou test also showed a promising potential for EC detection by genetic analyses of circulating tumor DNA (called PapSEEK) [37], further demonstrating that minimally invasive cervicovaginal sampling has immense potential for detecting EC and other uterine conditions [38].

In this study, we identified several biomarkers in CVL samples highly associated with EC using biomarker discovery and machine learning approaches. Key protein markers included: an immune checkpoint protein, TIM-3; two growth factors, VEGF and TGF-α; and an anti-inflammatory cytokine, IL-10. Other identified proteins, such as CA19–9, CA125, CYFRA 21-1, IL-15, MDC, MCP-1, MCP-3, PD-L2, TRAIL, can also be prioritized in the future, likely in combinations for specific and sensitive detection of EC, as we have demonstrated in our study using a logistic regression model. In addition, we explored associations of tested biomarkers with tumor characteristics, such as tumor size, presence and depth of myometrial invasion, and mismatch repair (MMR) protein expression, revealing a potential value of these selected biomarkers for clinical prognosis and/or monitoring therapies.

TIM-3 has been shown to be expressed by a variety of malignancies, including EC [39], and is a promising target for emerging immunotherapies [40]. Herein, we observed a gradual increase in TIM-3 levels in CVL samples collected from patients with endometrial hyperplasia and EC, suggesting it could be utilized for early disease detection. Previously, we also reported elevated levels of TIM-3 in CVL samples collected from patients with invasive cervical carcinoma [41]. This implies that combinations of cervicovaginal biomarkers likely need to be employed to distinguish different gynecologic malignancies. In addition, TIM-3 could be a potential target for immunotherapy in EC patients. Indeed, anti-TIM-3 antibodies have been recently tested in combination with other immune checkpoint inhibitors targeting PD-L1, in a phase 1b clinical trial, in patients with advanced MMR-deficient solid tumors, including EC, and have shown promising clinical utility [42]. Furthermore, since we observed a correlation of TIM-3 levels with tumor size and depth of myometrial invasion, TIM-3 might also serve as prognostic biomarker, similarly to other malignancies. Notably, TIM-3 has been associated with advanced disease and decreased survival in patients with cervical [43], ovarian [44], gastric [45], colorectal [46], renal [47] or hepatocellular [48] carcinomas.

Another identified biomarker, which has potential utility not only as a diagnostic marker but also as a target for therapy or clinical prognosis, is VEGF. Herein, we observed cervicovaginal VEGF to be elevated in patients with EC, and, similar to TIM-3, also to be correlated with tumor size and depth of myometrial invasion. Previously, VEGF has been evaluated as a prognostic marker in tissue and serum with mixed results [49]. In addition, anti-VEGF treatments alone or in combination with other immunotherapies have been evaluated in patients with advanced EC [50, 51]. These novel treatments specifically target MMR-proficient or microsatellite-stable tumors [51], which exhibit low response rates to monotherapies with PD-L1 inhibitors [52]. Intriguingly, our data revealed that patients with MMR-proficient tumors exhibit lower cervicovaginal levels of VEGF than patients with MMR-deficient tumors. Further investigation in the role of VEGF in the context of MMR status or microsatellite instability is warranted.

The epidermal growth factor, TGF-α, is also overexpressed in endometroid carcinoma compared to healthy endometrial tissues [53]. Furthermore, the expression of TGF-α has been associated with the progression of EC [54], which is in accordance with our findings in CVL. In the present study, we did observe a gradual increase in TGF-α levels in patients with endometrial hyperplasia and EC, as well as a positive correlation of TGF-α levels with deeper myometrial invasion within EC patients. Thus, TGF-α, together with other identified cervicovaginal markers, might have a great potential for detection of both endometrial hyperplasia and cancer, and possibly used as prognostic marker for EC recurrence, poor prognosis and/or response to therapies.

Finally, we identified an anti-inflammatory cytokine IL-10 to be associated with EC and endometrial hyperplasia. Another study also showed increased serum levels of IL-10 in EC patients [55]. Yet, the role of this pleiotropic cytokine in the pathophysiology of EC is not well understood. In the tumor microenvironment, IL-10 is required for proper T cell function, immune surveillance, and suppression of cancer-associated inflammation [56]. Consequently, there is a growing interest to exploit this key cytokine for development of new anti-cancer therapies. For example, blockade of IL-10 showed anti-tumor effect in organotypic cultures of human colorectal cancer liver metastases [57]. Accordingly, in the future, targeting IL-10 might be another tool in a portfolio of personalized immunotherapies for advanced or recurrent EC.

Overall, the unique aspects of our study include applying minimally invasive CVL sampling and multiplex immunoassay technology to EC biomarker discovery and use of an ethnically diverse cohort of women to study a broad range of cervicovaginal protein markers to identify robust diagnostic candidates for detection of EC. Though, our study has some limitations. We acknowledge that there were significant differences in age, menopausal status, and BMI in our cohort between the EC and benign control groups. Thus, our statistical models were adjusted for these factors. Although we excluded women currently menstruating from the study, we did not adjust the data for menstrual cycle day. Owing to a small sample size of patients with endometrial hyperplasia, we were not able to successfully apply some methods for biomarker discovery related to hyperplasia (e.g., ROC or logistic regression). The presented data related to this group was exploratory. Finally, our study design focused on patients undergoing hysterectomy, thus our control group consisted of patients diagnosed with benign gynecologic conditions, such as uterine leiomyoma, adenomyosis, and endometriosis. Although this approach allowed us to identify biomarkers discriminating EC from benign conditions, future investigations should be expanded to include healthy populations not undergoing hysterectomy. In this study, we tested physician-collected CVL samples using multiplex immunoassay technology; however, future studies may utilize self-collection devices [58–60] and rapid biomarker tests [61] to further expand and develop non-invasive EC diagnostics that are globally available.

## Conclusions

Our study provides a proof-of-principle that protein biomarkers associated with EC are present in the lower female reproductive tract and can be quantified via minimally-invasive CVL sampling. We identified several biomarker candidates discriminating patients with EC and patients with benign gynecologic conditions. Using a cross-validation approach, we demonstrated high accuracy of the combination of these biomarkers for EC detection. Future studies are warranted to validate and integrate our findings into predictive models using additional age-matched cohorts of women with low-grade EEC, more aggressive other EC subtypes, precancerous endometrial hyperplasia, and healthy controls, as well as self-collection methodologies (e.g., menstrual cups or self-collected lavages). Overall, improving early EC detection/diagnosis among diverse populations by developing cost-effective, robust, non-invasive diagnostics will decrease health inequities and positively impact women’s health.

## Supplementary Information

**Additional file 1: Supplementary Tables.** **Table S1.** Patient demographics and characteristics. **Table S2.** The significance of difference in comorbidities among the disease groups. **Table S3.** The significance of difference between protein levels among the disease groups.

**Additional file 2: Supplementary Figures. Fig. S1.** Differences in the first two principal components (PC1 and PC2) among the disease groups, menopausal status, and BMI categories. **Fig. S2.** ROC curves of biomarkers discriminating endometrial cancer and benign conditions. **Fig. S3.** Biomarkers discriminating endometrial cancer histological subtypes. **Fig. S4.** Data on tumor size and depth of myometrial invasion for endometrial cancer patients. **Fig. S5.** Cervicovaginal levels of proteins in all endometrial cancer patients stratified based on the tumor characteristics. **Fig. S6.** Correlations between cervicovaginal levels of protein biomarkers and tumor size. **Fig. S7.** Correlations between cervicovaginal levels of protein biomarkers and depth of myometrial invasion.

## Data Availability

The authors declare that the data supporting the findings of this study are available within the paper and its supplementary files. Additional data are available from the corresponding author upon reasonable request.

## Abbreviations

AUC: Area under the curve
BMI: Body mass index
CVL: Cervicovaginal lavage
EC: Endometrial cancer
EEC: Endometrial endometrioid carcinoma
MMR: Mismatch repair
ROC: Receiver operating characteristics
