# Supplementary material for: Protein biomarkers in cervicovaginal lavages for detection of endometrial cancer
Source: Biomark Res. 2022 Dec 2;10:88. doi: 10.1186/s40364-022-00438-5 (PMC9717501; doi:10.1186/s40364-022-00438-5)
Supplement: Supplementary file 2 — Additional file 2: Supplementary Figures. Fig. S1. Differences in the first two principal components (PC1 and PC2) among the disease groups, menopausal status, and BMI categories. Fig. S2. ROC curves of biomarkers discriminating endometrial cancer and benign conditions. Fig. S3. Biomarkers discriminating endometrial cancer histological subtypes. Fig. S4. Data on tumor size and depth of myometrial invasion for endometrial cancer patients. Fig. S5. Cervicovaginal levels of proteins in all endometrial cancer patients stratified based on the tumor characteristics. Fig. S6. Correlations between cervicovaginal levels of protein biomarkers and tumor size. Fig. S7. Correlations between cervicovaginal levels of protein biomarkers and depth of myometrial invasion. [file 40364_2022_438_MOESM2_ESM.pdf]

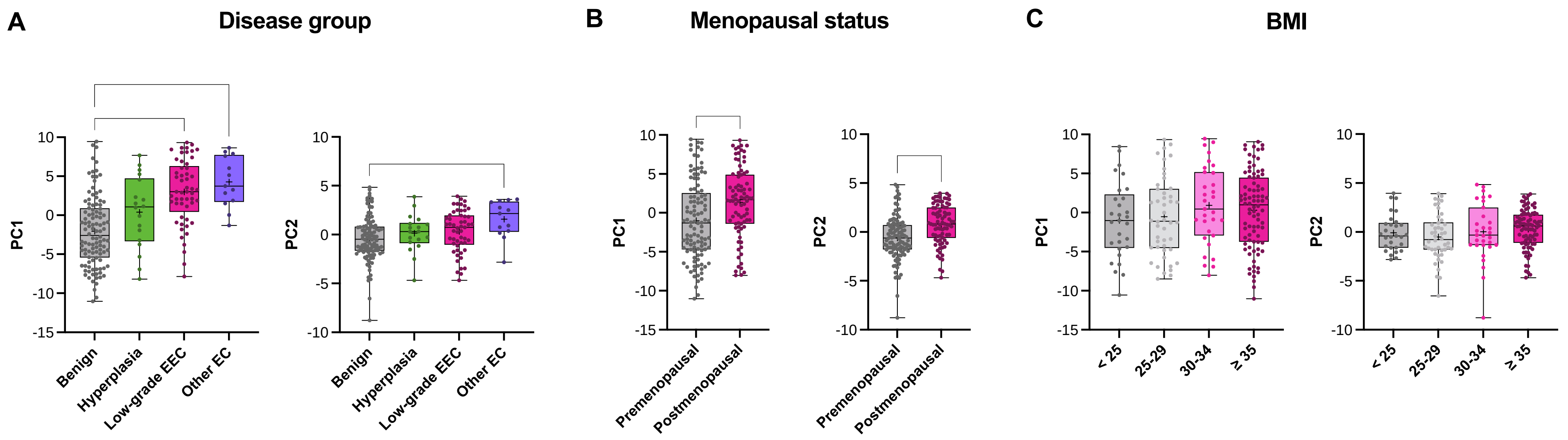

Figure S1. Differences in the first two principal components (PC1 and PC2) among the disease groups, menopausal status, and BMI categories. A principal component analysis (PCA) was performed using concentrations of 72 proteins in cervicovaginal lavages (*n*=192). The significant differences in PC1 and PC2 among the disease groups (**A**), menopausal status (**B**), and BMI categories (**C**) were assessed using an analysis of variance (ANOVA) with Tukey adjustment or unpaired two-tailed *t*-test. Asterisks indicate *P* values (* *P*<0.05; ** *P*<0.01; **** *P*<0.0001).

A Biomarkers discriminating low-grade EEC or other EC subtypes from benign conditions

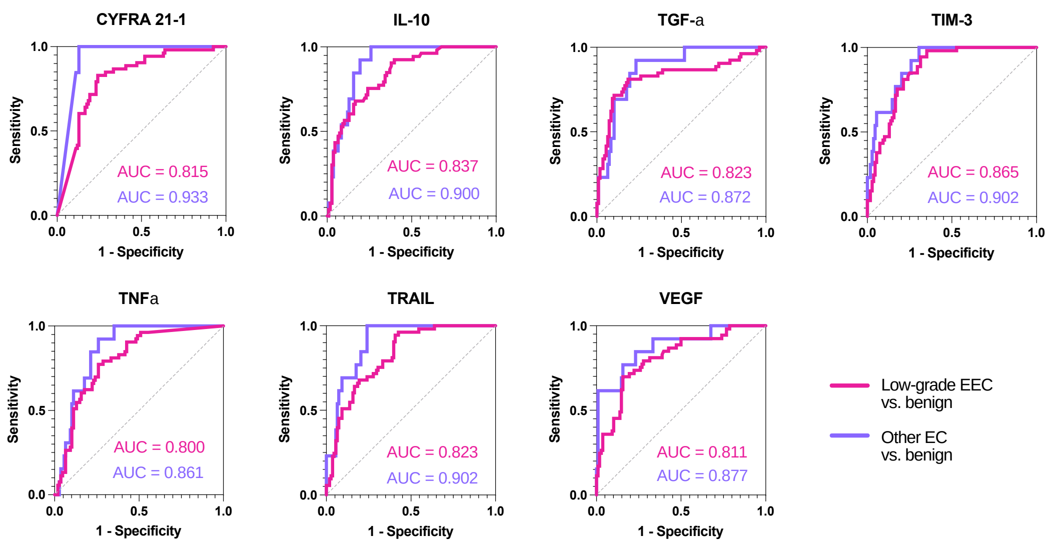

B Biomarkers discriminating other EC subtypes from benign conditions

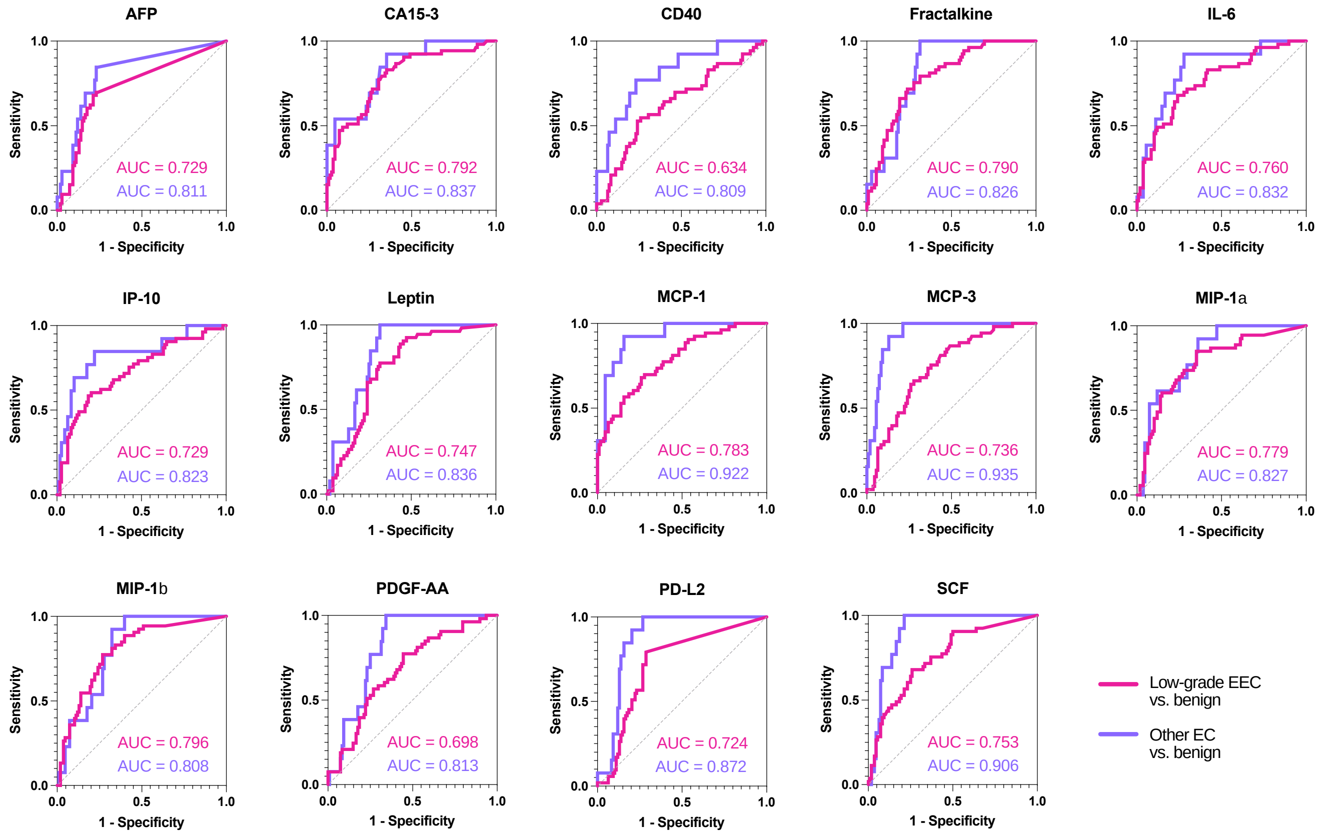

C Biomarkers discriminating other EC subtypes from low-grade EEC

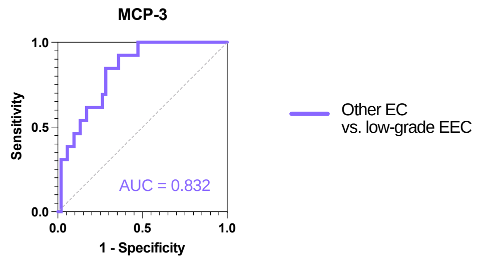

AUC:	Discriminatory potential:
> 0.900	Excellent
0.800 – 0.899	Good
0.700 – 0.799	Fair
< 0.700	Poor

Figure S2. ROC curves of biomarkers discriminating endometrial cancer and benign conditions. The receiver operating characteristics (ROC) analysis was used to identify biomarkers discriminating endometrial endometroid carcinoma (EEC) and other endometrial cancer (EC) types from benign conditions (**A-B**), as well as biomarkers discriminating EC histological types (other EC vs. low-grade EEC) (**C**). The area under the curve (AUC) was reported for each tested protein. ROC curves indicate 1 – specificity (x axis) and sensitivity (*y* axis). Proteins with the area under AUC greater than or equal to 0.8 or 0.9 were considered as good or excellent discriminators, respectively. Seven proteins exhibited good or excellent discriminatory properties for both EC types when compared to benign conditions (**A**). Additional 14 proteins had good or excellent discriminatory properties for other EC but not for low-grade EEC when compared to benign conditions (**B**). One protein, MCP-3, was a good discriminator for EC histological types (**C**).

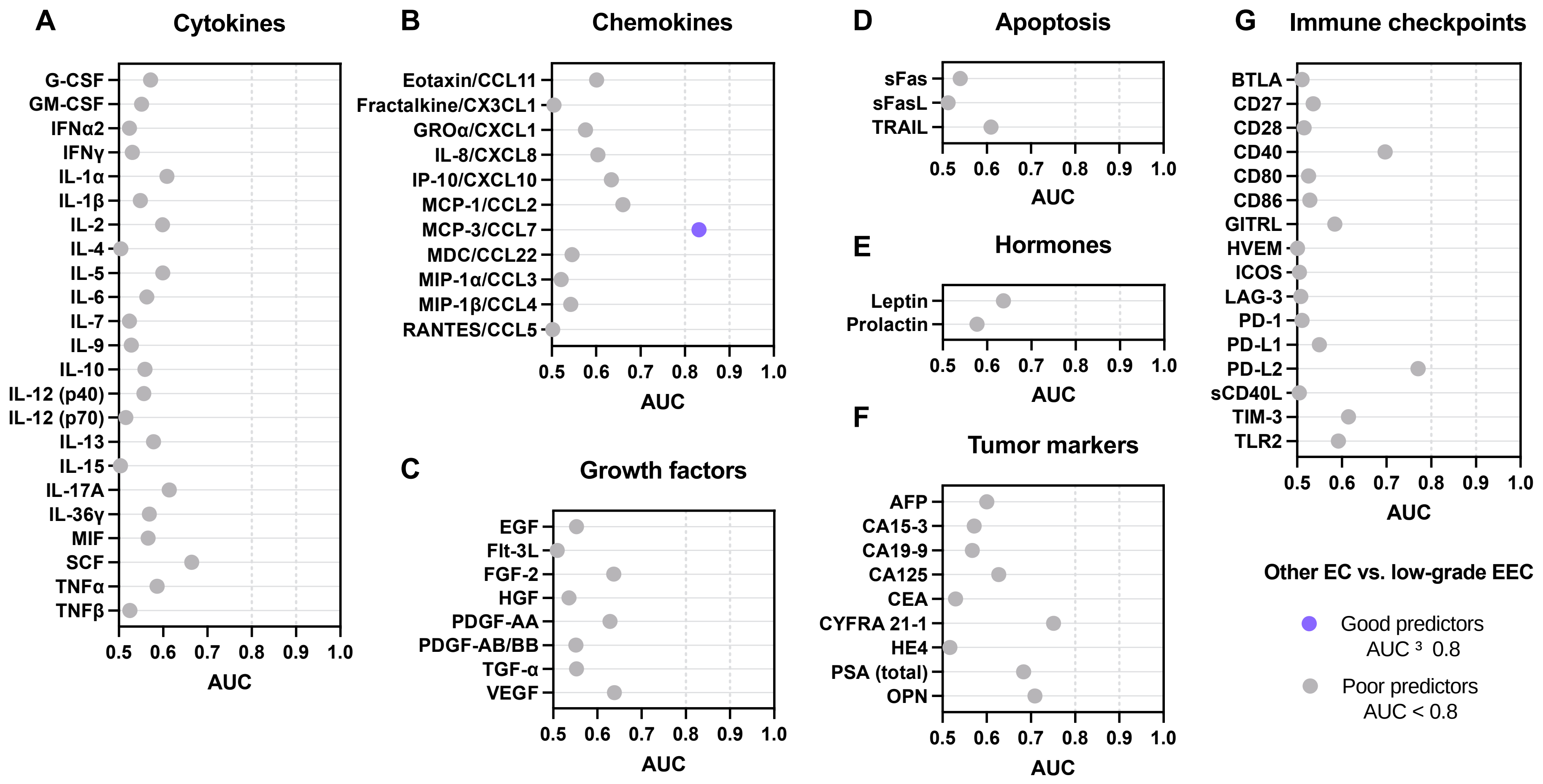

Figure S3. Biomarkers discriminating endometrial cancer histological subtypes. Cervicovaginal biomarkers discriminating other endometrial cancer (EC) subtypes from low-grade endometrial endometrioid carcinoma (EEC) were identified using the receiver operating characteristics (ROC) analysis. The area under the curve (AUC) was reported for each tested protein, including cytokines (**A**), chemokines (**B**), growth factors (**C**), apoptosis-related proteins (**D**), hormones (**E**), tumor markers (**F**), and immune checkpoint proteins (**G**). The strength of the discriminators was measured with area under the curve (AUC) values. Proteins with AUC greater than or equal to 0.8 were considered as good discriminators. A chemokine, MCP-3, was identified as a good discriminator between other EC and low-grade EEC.

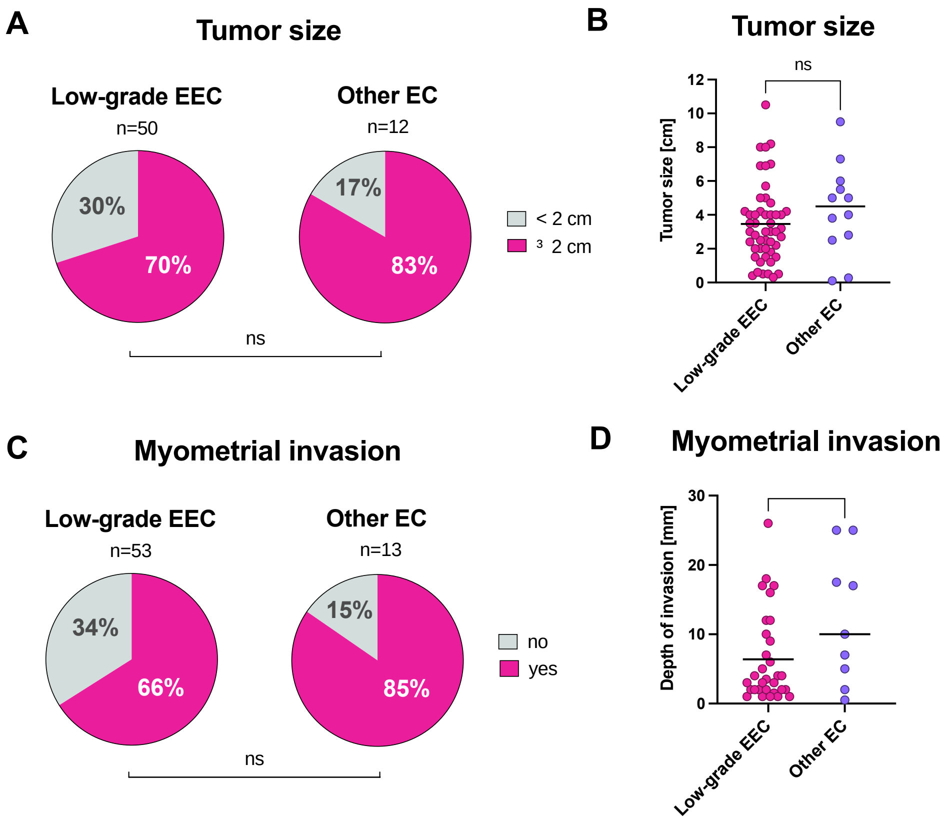

Figure S4. Data on tumor size and depth of myometrial invasion for endometrial cancer patients. Data for low-grade endometrioid carcinoma (EEC) and other endometrial cancer (EC) types were extracted from pathology reports. Data on tumor size were available for 62 out of 66 patients diagnosed with EC. Data on presence or absence of myometrial invasion were available for all EC patients (*n*=66). Data on depth of myometrial invasion were available for 40 EC patients. Pie charts show distribution of smaller (≤2 cm) and bigger (>2 cm) tumors (**A**), as well as proportion of presence of the myometrial invasion (**B**), among low-grade EEC and other EC. There was no significant difference (ns) in distribution of these tumor characteristics between EC histological types (calculated by Fisher's exact test). Scatter dot plots show the tumor size (measured in cm) (**C**) and the depth of myometrial invasion (measured in mm) (**D**) for the low-grade EEC and other EC. A horizontal line indicates a mean and asterisks indicate *P* values (* $P<0.05$). There was no significant (ns) difference between mean tumor sizes among the EC histological types. However, within tumors with myometrial invasion (*n*=40), other EC subtypes had deeper myometrial invasion compared to low-grade EEC. The significant differences were assessed using unpaired two-tailed *t*-test.

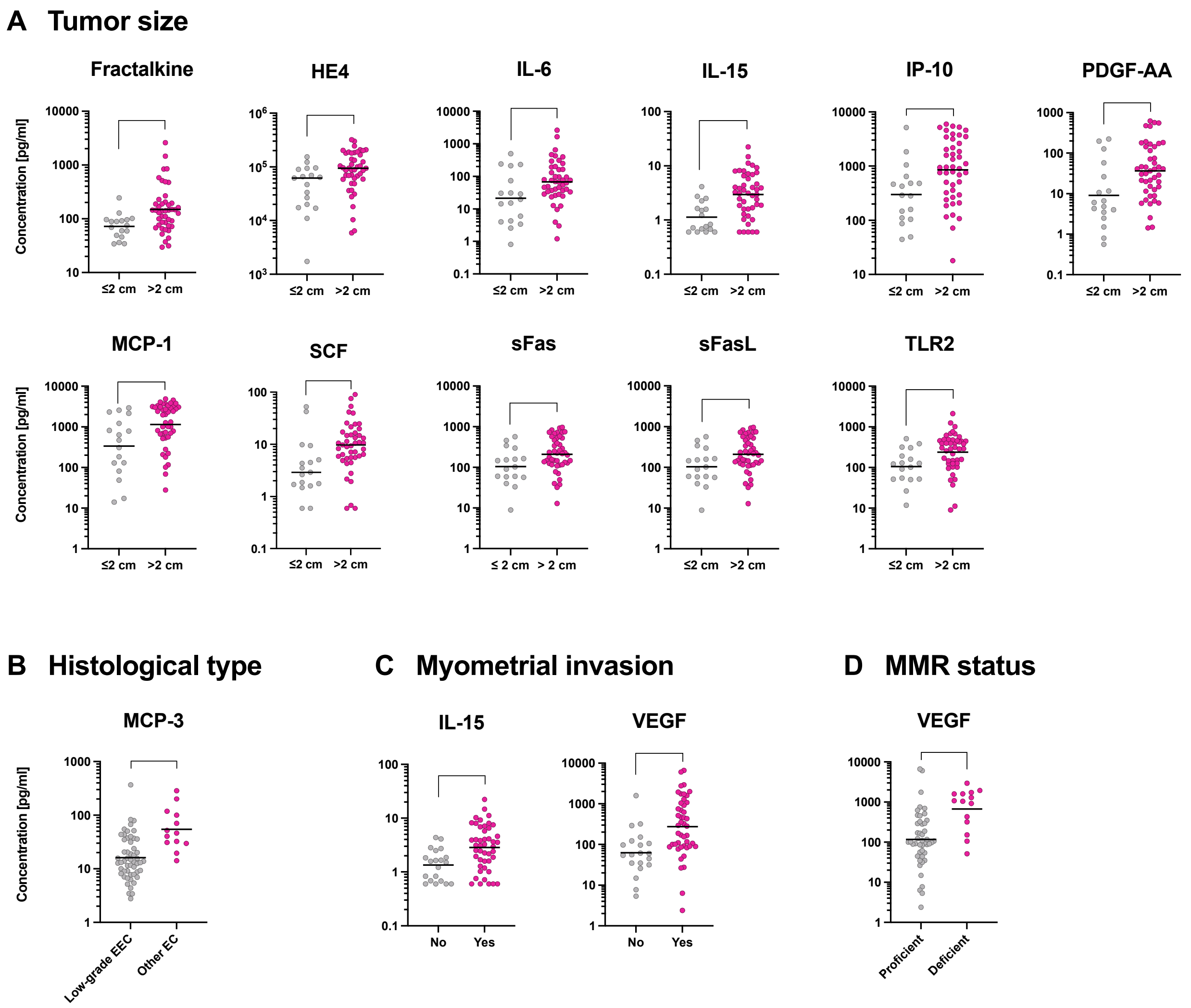

Figure S5. Cervicovaginal levels of proteins in all endometrial cancer patients stratified based on the tumor characteristics. A volcano plot analysis was used to assess differences in the protein levels among patients with all endometrial cancer stratified based on tumor characteristics, such as tumor size (≤2 cm vs. >2 cm) (**A**), histological type (low grade endometrial endometroid carcinoma (EEC) vs. other endometrial cancer (EC) types) (**B**), presence of myometrial invasion (no vs. yes) (**C**), and mismatch repair (MMR) protein status (MMR-proficient vs. MMR-deficient) (**D**). Statistical significance was determined using multiple *t*-test with the false discovery rate (FDR) correction. Proteins with $q<0.01$ were considered significant. Scatter dot plots show concentrations of identified protein biomarkers in individual samples. A horizontal line indicates the mean and asterisks indicate *P* values (* $P<0.05$; ** $P<0.01$; *** $P<0.001$; **** $P<0.0001$).

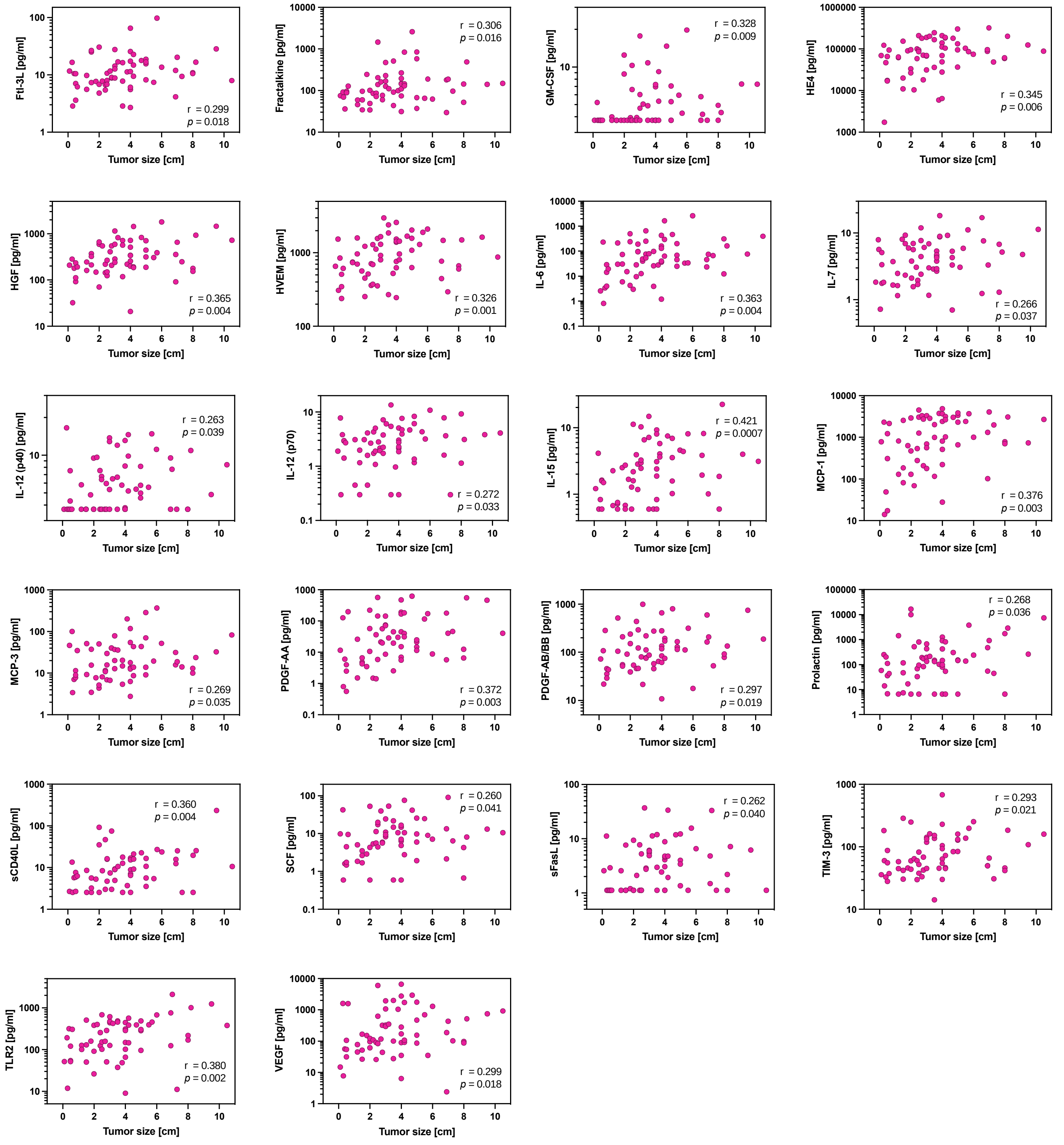

Figure S6. Correlations between cervicovaginal levels of protein biomarkers and tumor size. Scatterplots show the relationship between concentrations of proteins (log-transformed pg/ml) and the size (measured in cm) of all endometrial tumors, including low-grade endometrial endometrioid carcinoma and other subtypes (data available for *n*=62). Correlation coefficients (r) were calculated using Spearman's rank correlation analysis. *P* values were calculated using two-tailed test. Scatterplots represent only biomarkers with *P*<0.05. Levels of 22 proteins positively correlated with the tumor size.

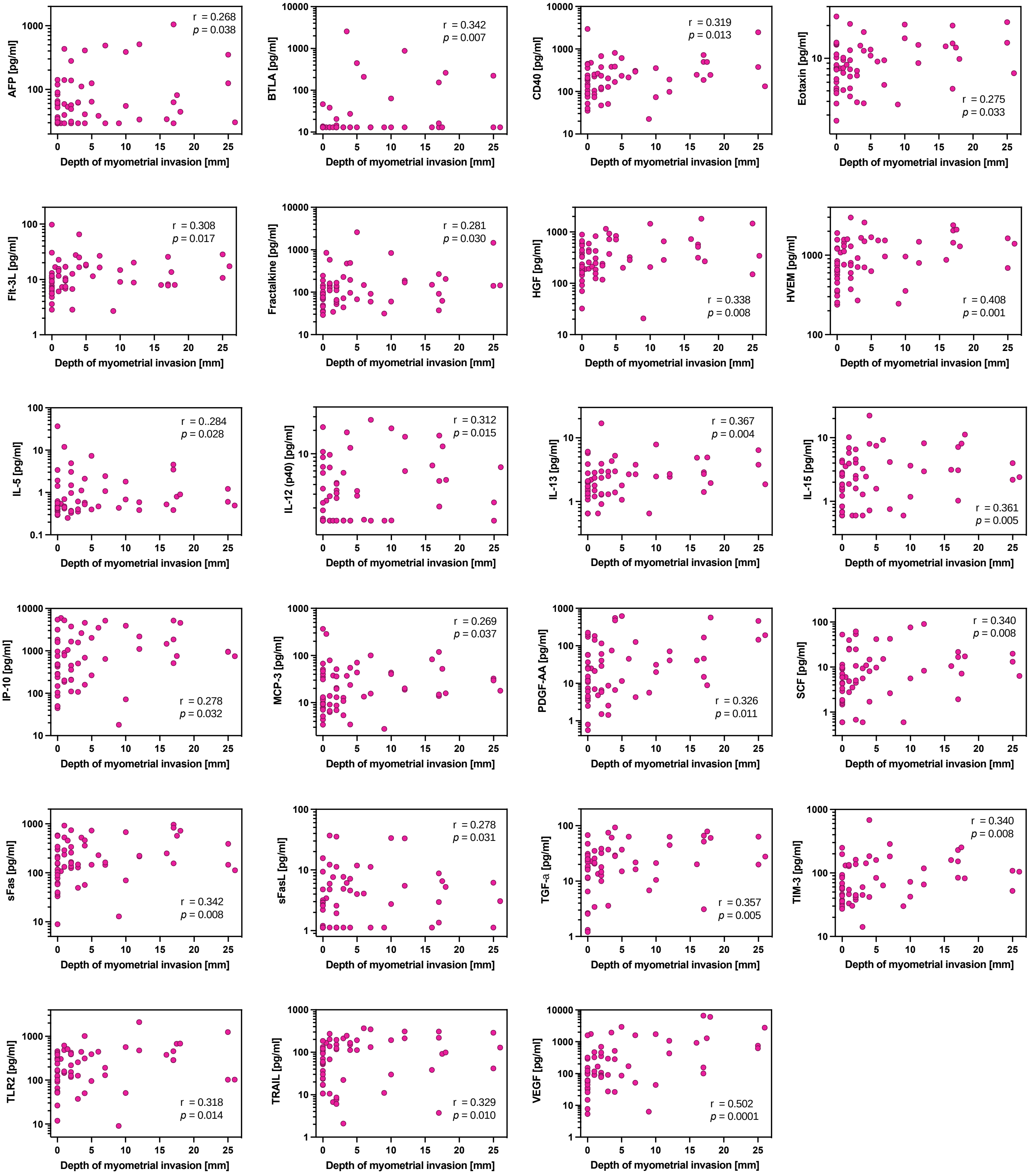

Figure S7. Correlations between cervicovaginal levels of protein biomarkers and depth of myometrial invasion. Scatterplots show the relationship between concentrations of proteins (log-transformed pg/ml) and the depth of myometrial invasion (measured in mm) in all endometrial tumors, including low-grade endometrial endometrioid carcinoma and other subtypes (data available for *n*=60). Correlation coefficients (r) were calculated using Spearman's rank correlation analysis. *P* values were calculated using two-tailed test. Scatterplots represent only biomarkers with *P*<0.05. Levels of 23 proteins positively correlated with the depth of myometrial invasion.
